# Proceedings of the Chicago Medical Society

**Published:** 1868-12-01

**Authors:** 


### PROCEEDINGS OF THE CHICAGO MEDICAL SOCIETY.

During the past few months, the meetings of this Society have been held but once in four weeks. Hereafter they will be held weekly. The following are instructive cases, with the report of which pathological specimens were presented:

### REMOVAL OF A LARGE OVARIAN TUMOR — RECOVERY.

Prof. Powell presented an unusually large ovarian tumor, which he had removed from a patient, thirty years of age. The patient first observed a slight swelling of the abdomen, about a year ago, the growth of which, however, did not cause her special inconvenience till January last. From this time to the day of the operation, August 31st, the abdomen became very rapidly distended. Paracentesis had quite recently been twice performed by other physicians, with the discharge of very little fluid. The tumor was removed through an opening in the abdomen, extending from the ensiform cartilage to the symphysis pubis. Before the tumor could be brought through the opening, it was necessary to evacuate large quantities of fluid from several cysts, and to break up with the hand several extensive adhesions. After the peduncle was firmly tied with a strong linen cord, it was divided and returned within the abdomen. The external wound was closed by means of twisted sutures, and supported by a flannel bandage. The tumor, with the serum contained in its cysts, weighed sixty pounds. The patient made a rapid recovery with scarcely any marked excitement of the circulation, diminution of appetite, or loss of sleep.

### CANCER OF THE STOMACH.

Dr. Bogue exhibited the stomach of a male patient, 69 years of age, who had never, with scarcely any exception, suffered from sickness, till a year before his death. He first experienced slight symptoms of dyspepsia. There was gradual loss of appetite, with emaciation. Four months previous to death, there was first observed a small tumor, with tenderness, in the region of the stomach. The symptoms increased in severity, although at no time was there much acute pain or much vomiting. On the tenth of July he was wholly unable to swallow. From this time to the day of his death, August 29th, he was nourished by nutritive enemata. At the autopsy, the stomach was found very much thickened and contracted, with a very narrow passage through it, and almost no trace of mucous membrane. The other organs of the body were from a similar disease.

### CONCRETION FROM THE FEMALE URETHRA.

Prof. Miller exhibited a small concretion, one-third of an inch long, and one-half an inch in circumference, which he had found impacted in the urethra of a female twenty-five years of age. It was peculiarly grooved in such a manner that the urine could slowly escape through it, even when the mucous membrane was constricted around it. The case was interesting from the fact that it had been under the care of three different physicians, who had treated it as inflammation of the bladder, or as uterine disease.

### RAPID INFILTRATION OF A LUNG WITH TUBERCULOUS MATTER.

Prof. Ross exhibited the left lung of a young man who died at the Cook County Hospital. The case was most remarkable from the fact that the patient had been strong and rugged till eight weeks before death. He died two weeks after entering the hospital, with the characteristic emaciation, hectic night sweats, and expectoration. There was almost no respiratory motion on the left side. The left lung was found almost completely solidified with tuberculous deposit. There were large cavities at the apex.

Prof. Ross also presented a typical specimen of "hob-nail liver." The organ was covered with the characteristic nodules, and weighed but one and three-fourth pounds. The patient, a female aged twenty-five years, had been of intemperate habits. Death occurred two weeks after the patient entered the hospital, with all the symptoms of typhoid fever. The characteristic lesions of this disease were found in the ilium.

### SARCOMA OF THE CHOROID.

Dr. Holmes exhibited an eye which he had recently removed from a patient at the Chicago Charitable Eye and Ear Infirmary. There was a well defined tumor occupying the external and lower portion of the globe, and extending from very near the ciliary muscle to the optic nerve. At the first examination, three weeks previous to the extirpation, the tumor could be readily seen by concentrated light, and still better with the ophthalmoscope. There was no perception of light except at the inner and upper portion of the retina. The pupil, of normal size, acted quite freely under the influence of light. There had been no pain. The globe was not hard on palpation. The cornea had not lost its sensitiveness, although an increase of the intraocular tension and loss of sensibility of the cornea are very frequent symptoms of choroidal tumor. There was no trembling of the retina, which is usually observed when there is detachment of the retina. It was nearly a year since the patient first observed any impairment of vision. Two days before entering the Infirmary, the patient presented himself, suffering excruciating pain in and around the eye, with much redness and œdema of the conjunctiva and hæmorrhage behind the lens. These symptoms had appeared very suddenly. A horizontal section of the globe after its removal revealed a sarcomatous tumor of the choroid, situated as above described. The central portions consisted of a soft, pulpy, yellowish red mass, surrounded by a layer of black tissue, nearly a line in thickness. Dr. Lyman made a microscopic examination of the specimen, and states that the tumor is sarcomatous, with characteristics as described on page 460 of the translation of Stellwag. The outer portion of the tumor is dark from the presence of black pigment in many of the cells.

